# Genomic Surveillance of 3R Genes Associated with Antibiotic Resistance in *Mycobacterium tuberculosis* Isolates from Kazakhstan

**DOI:** 10.3390/antibiotics15010026

**Published:** 2025-12-30

**Authors:** Savva Timochshuk, Aldan Shamukhan, Bakhtiyar Yakupov, Dana Auganova, Ulan Zein, Aigerim Turgimbayeva, Pavel Tarlykov, Sailau Abeldenov

**Affiliations:** 1 National Center for Biotechnology, Astana 010000, Kazakhstan; 2 Department of General Biology and Genomics, L. N. Gumilyov Eurasian National University, Astana 010000, Kazakhstan

**Keywords:** *Mycobacterium tuberculosis*, drug resistance, 3R genes, Kazakhstan

## Abstract

Background/Objectives: Multidrug-resistant tuberculosis remains a critical public health challenge in Kazakhstan, yet the genomic determinants contributing to its emergence are still insufficiently understood. Although the quantity of genomic studies from Central Asia and the wider post-Soviet region has increased in recent years, the involvement of DNA repair and genome maintenance pathways in the development of resistance within Kazakhstan has not been comprehensively explored. Methods: In this study, we performed whole-genome analysis of 175 *Mycobacterium tuberculosis* clinical isolates collected across Kazakhstan between 2010 and 2022 to evaluate the contribution of single-nucleotide polymorphisms in DNA replication, repair, and recombination (3R) genes to the evolution of drug resistance. Results: Alongside well-established resistance mutations in *gyrA*, we identified recurrent variants in 3R-associated loci (genes involved in DNA replication, repair, and recombination)—including *polA*, *uvrC* and *ligC*—that were enriched among drug-resistant isolates, suggesting a broader role for genome maintenance pathways in facilitating resistance evolution under treatment pressure. Conclusions: These findings provide the first region-specific genomic insights into 3R gene variation in Kazakhstani *M. tuberculosis* isolates.

## 1. Introduction

Tuberculosis (TB), caused by *Mycobacterium tuberculosis* (Mtb), remains a leading cause of infectious mortality worldwide, with an estimated 10.7 million incident cases and 1.23 million deaths in 2024 alone, underscoring its status as a persistent global health crisis exacerbated by diagnostic delays and underfunding [1,2]. The advent of multidrug-resistant (MDR-TB) and extensively drug-resistant (XDR-TB) forms has intensified this burden, with approximately 390,000 new MDR/RR-TB (rifampicin-resistant tuberculosis) cases reported globally in 2024, driven by inadequate treatment adherence, poor infection control, and the pathogen’s remarkable adaptability [1,3]. Kazakhstan exemplifies this challenge, ranking among the 30 high MDR-TB burden countries for 2021–2025 [4,5,6,7]. One contributing factor is the widespread presence of the Beijing lineage, which is associated with increased transmissibility and a higher likelihood of drug resistance. These biological factors intersect with broader social and economic challenges [8,9].

In contrast to pathogens such as *Klebsiella pneumoniae* and *Escherichia coli*, which commonly acquire antimicrobial resistance through horizontal transfer of extended-spectrum β-lactamase (ESBL) and other resistance determinants carried on mobile plasmids that can move between strains and even across species, *Mycobacterium tuberculosis* follows a fundamentally different evolutionary path. Because *M. tuberculosis* lacks plasmids and engages in minimal recombination, it cannot gain resistance via horizontal gene transfer; instead, drug resistance arises solely through spontaneous chromosomal mutations—predominantly SNPs, small insertions or deletions, and occasional larger structural variants—that occur during replication and become fixed under selective pressure from inadequate or interrupted therapy. As a result, whereas resistant healthcare-associated infections in plasmid-bearing bacteria may emerge through rapid plasmid dissemination or clonal spread, *M. tuberculosis* accumulates resistance mutations slowly and clonally, at a modest baseline mutation rate (0.3–0.6 SNPs per genome per year), ultimately enabling the stepwise emergence of MDR- and XDR-TB [10,11,12,13].

Drug-resistance in *Mycobacterium tuberculosis* arises through mutations that typically alter drug targets, prodrug activators or efflux regulators, thereby conferring resistance via mechanisms such as target modification or enzymatic inactivation. Although these mutations often impose a fitness cost, this burden can be mitigated through compensatory changes or favorable epistatic interactions that restore growth rates without reversing resistance. Indeed, epistasis among resistance-conferring and compensatory mutations, together with the broader genetic background of the strain, profoundly shapes the biology, persistence and epidemiology of drug-resistant TB. For instance, mycobacteria harboring both an *rpoB* mutation (rifampicin resistance) and a fluoroquinolone-resistance mutation in *gyrA* can display higher competitive fitness than strains carrying either mutation alone. Similarly, strains combining an *rpoB* mutation with a compensatory mutation in *RpoC* show enhanced fitness relative to those with only the *rpoB* mutation and exhibit an increased likelihood of acquiring additional resistance mutations, promoting the amplification of resistance. Collectively, these examples illustrate how epistatic interactions can reduce fitness costs and facilitate the evolution and spread of multidrug-resistant strains. A more comprehensive understanding of these relationships will be essential for strengthening antibiotic resistance surveillance and guiding the deployment of new TB treatment regimens [14,15,16].

The most prevalent chromosomal markers of resistance include mutations in *rpoB* for rifampicin, *katG* and *inhA* promoter for isoniazid, *embB* for ethambutol, *pncA* for pyrazinamide, *rpsL* and *rrs* for streptomycin, and *gyrA* for fluoroquinolones [17,18,19]. While these canonical loci account for the majority of phenotypic resistance, hypermutability driven by defects in DNA replication, repair, and recombination (3R) genes accelerates adaptation to antibiotics by elevating genome-wide mutation rates and increasing the probability of acquiring resistance-conferring variants in drug targets [20,21]. SNPs in 3R genes—such as those involved in base excision repair (BER), nucleotide excision repair (NER), or homologous recombination (HR)—are enriched in MDR/XDR isolates, particularly within Beijing lineage strains, promoting genetic diversity that facilitates survival under host oxidative stress and therapeutic pressure [20,22]. Investigating these variants can deepen our understanding of resistance evolution, identify predictive biomarkers for emerging MDR phenotypes, and reveal therapeutic targets within repair pathways to prevent mutational escape [21,23].

This study provides novel insight into the role of 3R genes in the emergence of drug resistance by analyzing SNP data from 175 Mtb isolates collected in Kazakhstan—a sentinel high-MDR/RR-TB region—focusing on the distribution and frequency of mutations across a predefined panel of 67 3R genes.

## 2. Results

### 2.1. Analysis of SNPs

Analysis of mutations in 3R genes among *Mycobacterium tuberculosis* strains resistant to various antimicrobial drugs revealed a pattern of non-synonymous variants potentially associated with resistance phenotypes (Table S1). Across the dataset, genes such as *gyrA* exhibited recurrent mutations linked to fluoroquinolone resistance, including Asp94Gly (observed in up to 92.3% of rifampicin-resistant strains and 87.5% of moxifloxacin-resistant strains at 0.25 mg/L) and Ala90Val (prevalent in 85.7% to 100% across multiple drug categories), consistent with established mechanisms of topoisomerase inhibition.

In broader DNA repair pathways, mutations such as Gly58Arg in *mutT2* were ob-served at high frequencies across multiple resistance profiles. Similarly, the Ile245Thr mutation in *recF*, involved in homologous recombination and replication fork restart, was prevalent (up to 100% in certain subsets, including 83.0% in isoniazid-resistant isolates). Further patterns emerged in excision repair components, with *uvrC* displaying Val434Ala and Val289Ile mutations at high frequencies (e.g., 78.8% in streptomycin-resistant isolates). In our cohort of antibiotic-resistant *Mycobacterium tuberculosis* isolates, nearly all strains exhibited mutations in *lig* genes (with frequencies reaching 93.9% in pyrazinamide-resistant subsets). Additionally, the Phe358Leu mutation in Rv2090, was highly frequent (up to 100% in subsets like bedaquiline-resistant isolates and 83.0% in isoniazid-resistant strains).

Similarly, mutations in the *dnaB* gene exhibited high prevalence (ranging from 76.2% to 83.0%) among isolates resistant to core first-line drugs, including isoniazid, rifampicin, and ethambutol. Examination of replication-associated genes, including *polA*, revealed prevalent mutations such as Arg188Gly and Thr186Pro, occurring at rates of 70.1% to 83.0% in strains resistant to isoniazid, rifampicin, and ethambutol.

**Figure 1:**
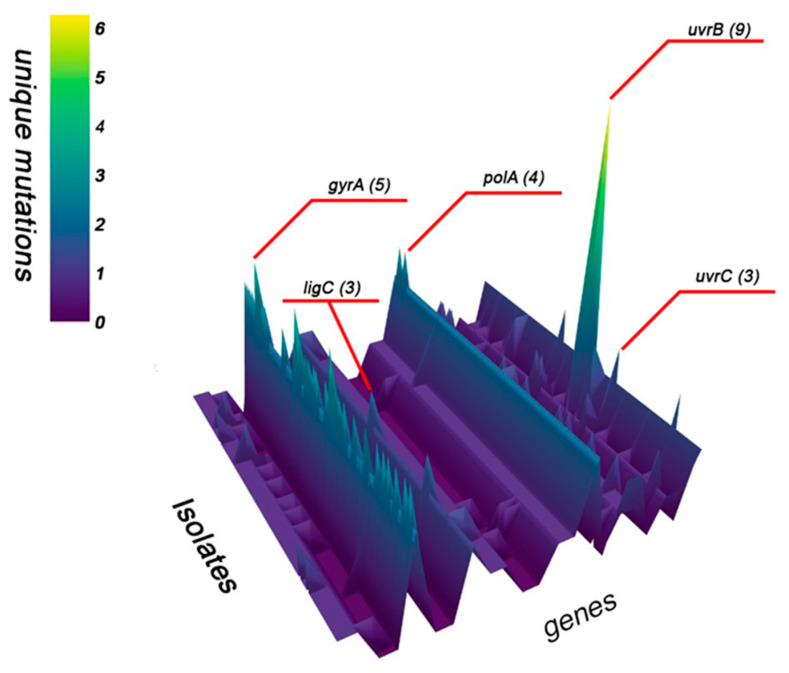
The distribution of mutations across the examined genes in 175 isolates. The extremes of the mutation count appear as peaks on the graph.

**Figure 2:**
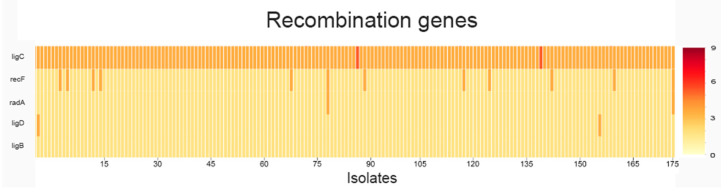
The distribution of mutations among recombination-associated genes.

**Figure 3:**
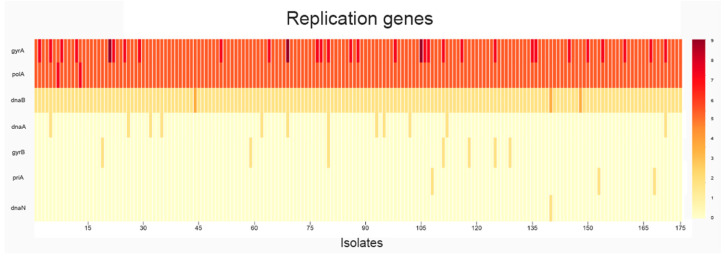
The distribution of mutations among replication-associated genes.

**Figure 4:**
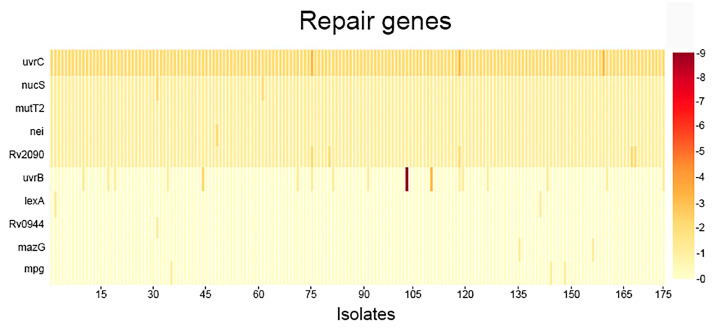
The distribution of mutations among repair-associated genes.

To provide a clear visualization of the distribution and frequency of single-nucleotide polymorphisms across the 67 targeted DNA replication, repair, and recombination (3R) genes in all 175 *Mycobacterium tuberculosis* isolates, we conducted a systematic analysis of mutation load per gene. This approach enabled the identification of highly variable loci as well as genes with rare but consistent mutations, potentially reflecting functional constraints or adaptive significance in the context of MDR phenotype emergence. The results of this analysis including the overall mutational profile, isolate-level distribution, and associations with clinical resistance patterns are presented (Figure 1, Figure 2, Figure 3 and Figure 4) (Tables S2 and S3).

The genes with the highest mutation counts are *uvrB* (9 mutations), *gyrA* (5 mutations), and *polA* (4 mutations). Although, *dnaB*, *nucS*, *ligD*, *ligB*, *mutT2*, *nei*, *radA*, *recF*, and Rv2090 each exhibit a single mutation in all isolates. Additionally, *ligC* and *uvrC* consistently show two mutations per isolate (Table S2).

The genes *gyrA*, *polA*, and *uvrB* (Figure 3 and Figure 4) appear to be under selective pressure and may play a significant role in the acquisition of antibiotic resistance, whereas *nucS*, *ligD*, and other DNA repair genes seem to be more conserved, suggesting they are less variable and less directly involved in resistance development. In addition to these novel DNA repair associations, our analysis identified known first- and second-line drug resistance determinants, validating the consistency of our findings with established resistance mechanisms.

### 2.2. Spectrum of Nucleotide Substitutions

**Figure 5:**
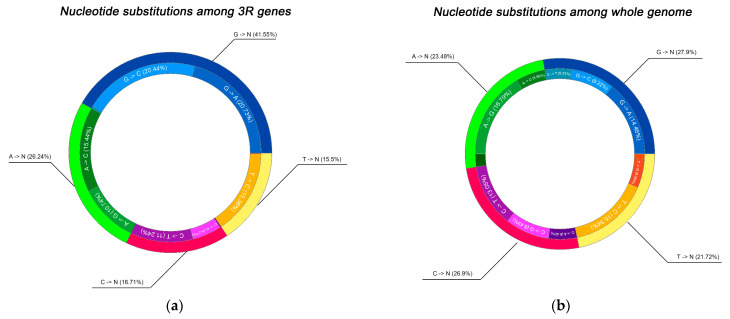
(**a**) Description of nucleotide substitutions among 3R genes; (**b**) nucleotide substitutions among whole genome.

To gain deeper insight into the molecular nature and potential drivers of the observed mutations, we systematically characterized the spectrum of nucleotide substitutions in two complementary contexts: first, within the 67 targeted DNA replication, repair, and recombination genes, and second, across the entire genome of the sequenced isolates for comparative reference. This parallel analysis was designed to reveal whether mutational signatures in DNA maintenance pathways exhibit distinct biases compared to genome-wide patterns, which could indicate pathway-specific selective pressures, heightened vulnerability to particular types of DNA damage or differential efficiency of repair mechanisms operating on these functionally critical loci (Figure 5a,b).

The analysis of base substitution patterns across all isolates showed that transitions involving guanine constituted the largest proportion of observed mutations. Together, G > C and G > A substitutions represented 41% of all recorded events in the dataset. Notably, transitions originating from thymine occurred less frequently, with T > C substitutions comprising 15.5% of the total. Within this group, G → C (20.4%) and G → A (20.7%) transitions were dominant, while G → T substitutions were rare (0.4%). Similarly, A → N transitions comprised 26% of all substitutions, primarily represented by A → C (15.4%) and A → G (10.7%), whereas A → T substitutions were nearly absent (0.1%). Mutations originating from cytosine and thymine were comparatively infrequent, including 16.7% C → T and 15.5% T → C transitions.

**Figure 6:**
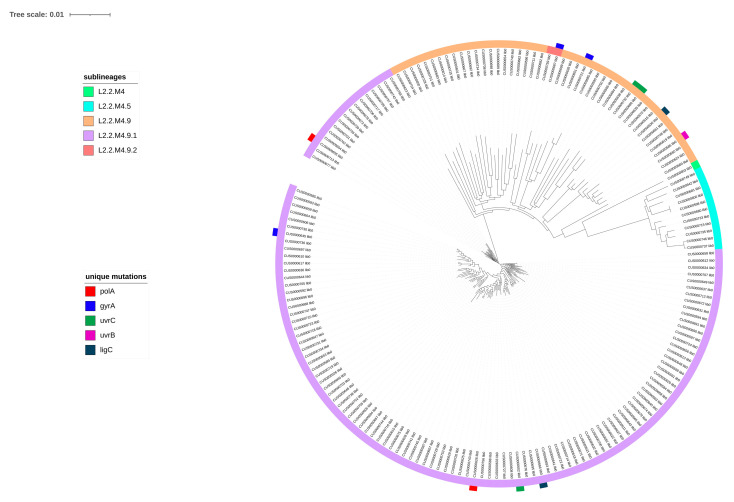
The phylogenetic reconstruction encompassing five sublineages—L2.2.M4, L2.2.M4.5, L2.2.M4.9, L2.2.M4.9.1, and L2.2.M4.9.2—reveals a set of sublineage-specific mutations distributed across several genes, including *polA*, *gyrA*, *uvrC*, *uvrB*, and *ligC*.

To address the relationship between lineage structure and mutation profiles, we constructed a phylogenetic tree representing five sublineages (L2.2.M4, L2.2.M4.5, L2.2.M4.9, L2.2.M4.9.1, and L2.2.M4.9.2) (Figure 6).

Analysis of the phylogenetic tree revealed distinct mutations across five genes among the isolates. Mutations in *gyrA* were detected in three isolates (CUS0000645 lib0, CUS0000687 lib0, CUS0000721 lib0), while *uvrC* mutations were also present in three isolates (CUS0000732 lib0, CUS0000686 lib0, CUS0000602 lib0). *polA* and *ligC* each harbored mutations in two isolates (*polA*: CUS0000655 lib0, CUS0000626 lib0; *ligC*: CUS0000630 lib0, CUS0000666 lib0), whereas *uvrB* exhibited a mutation in a single isolate (CUS0000586 lib0).

## 3. Discussion

The genomic analysis of 175 *Mycobacterium tuberculosis* isolates from Kazakhstan revealed a complex mutational landscape within genes responsible for DNA replication, repair, and recombination. These loci are essential for maintaining genomic integrity, yet they also serve as potential sources of variability that enable bacterial adaptation under antibiotic pressure [21,24,25]. The predominance of nonsynonymous substitutions in genes such as *gyrA*, *uvrB*, *recF*, and *polA* may suggest the influence of selective pressures potentially shaping the evolution of multidrug-resistant tuberculosis in high-burden settings (Table S4). The observation that *gyrA* exhibited recurrent mutations at codons 90 and 94, including Ala90Val and Asp94Gly, aligns with prior global reports linking these substitutions to fluoroquinolone resistance and impaired binding of DNA gyrase inhibitors [26,27,28]. These variants were more pronounced in strains resistant to moxifloxacin and levofloxacin, aligning with prior reports on *gyrA* alterations driving quinolone resistance in multidrug-resistant tuberculosis [29,30]. This consistency reinforces the idea that drug-driven selection continues to shape the *M. tuberculosis* genome across geographically diverse populations [1]. The accumulation of mutations within *gyrA* and other repair-related loci underscores the intimate interplay between mutagenesis and adaptation in *M. tuberculosis*. Collectively, these findings establish a critical link between compromised DNA repair fidelity and the evolution of multidrug-resistant and extensively drug-resistant tuberculosis, suggesting that early detection of variants in 3R genes could serve as valuable diagnostic markers to guide therapeutic decisions and mitigate the emergence of resistance [20,31]. Notably, one study [29] also mentioned a substitution at codon 95, although it was reported at a low frequency. In contrast, within our cohort, mutations at codon 95 were more frequently observed. Moreover, we additionally detected recurrent substitutions at codons 21 and 668 (Table S3). However, despite their repeated occurrence, we cannot confidently propose these positions as reliable resistance markers: in larger international datasets, their prevalence for several antibiotics does not exceed 95%, and in instances where these substitutions appear more common, the evidence is limited to only a few isolates, restricting interpretability.

While mutations were indeed detected in DNA repair genes such as *mutT2*, *recF*, and *uvrC* among the analyzed isolates, potentially indicating broader disruptions in repair fidelity that could indirectly support the emergence and persistence of antibiotic resistance, direct causal links remain unsubstantiated for the specific variants identified in this study. For instance, the Gly58Arg substitution in *mutT2* is fixed in the Beijing sublineage of *M. tuberculosis* and serves solely as a phylogenetic marker, with no functional contribution to drug resistance. Although the Beijing genotype as a whole has been correlated with multidrug resistance in various global cohorts, this appears attributable to lineage-specific evolutionary pressures rather than the *mutT2* Gly58Arg mutation itself [32,33]. Similarly, while mutations in *recF* have been implicated in altered DNA recombination and potential contributions to hypermutator states in *M. tuberculosis*, the Ile245Thr variant observed here lacks documented evidence of such effects or direct ties to antibiotic resistance, distinguishing it from other reported *recF* alterations associated with drug tolerance [20]. These findings underscore the need for functional validation to clarify whether these SNPs confer hypermutator phenotypes, as previously described in *M. tuberculosis* and other pathogens, or merely reflect neutral genetic drift. In this context, the consistent detection of substitutions in *uvrC*, a key component of the nucleotide excision repair pathway, is particularly noteworthy. Defective *uvr* function can impair the excision of oxidatively damaged nucleotides, leading to an accumulation of mutational load and potentially facilitating resistance evolution [34,35,36]. The enrichment of *uvrC* variants in streptomycin- and isoniazid-resistant isolates observed in this study supports the hypothesis that defective excision repair indirectly promotes antibiotic tolerance by increasing mutational diversity.

Genes involved in DNA ligation and non-homologous end-joining (NHEJ) showed particularly high mutation burdens. Variants in the *lig* family were detected in nearly all resistant strains, reaching 93.9% prevalence in pyrazinamide-resistant subsets. Disruption of these genes is expected to impair ligation efficiency during NHEJ and other repair pathways, thereby increasing genomic instability and mutational supply for adaptation under drug pressure [37]. Similarly, the Phe358Leu substitution in Rv2090 (a putative accessory factor in repair epistasis) achieved near-fixation in bedaquiline-resistant isolates (up to 100%) and 83.0% in isoniazid-resistant strains, where it has been implicated in novel epistatic interactions that enhance genomic plasticity and resistance evolution [38].

Replication fork progression and fidelity were also frequently compromised. The replicative helicase gene *dnaB* harbored mutations in 76.2–83.0% of isolates resistant to isoniazid, rifampicin, and ethambutol. In *Escherichia coli*, analogous *dnaB* defects dramatically elevate mutation rates and accelerate acquisition of rifampicin resistance [39]; however, no comparable evidence has yet been reported for *Mycobacterium tuberculosis*, and our findings therefore raise the possibility—still unconfirmed—of a similar mechanism operating in clinical strains. Likewise, *polA* (DNA polymerase I) exhibited recurrent nonsynonymous changes (e.g., Arg188Gly and Thr186Pro) at frequencies of 70.1–83.0% in the same resistance categories. Although these specific *polA* variants have not been functionally characterized in *M. tuberculosis*, engineered error-prone *polA* alleles in experimental systems have been shown to increase mutation rates in drug-target genes such as *rpoB* and *atpE* [40]. Further validation in *M. tuberculosis* would require targeted functional assays—such as assessing rifampicin mutation frequency in strains engineered to carry these *polA* substitutions—to determine whether they contribute to elevated mutational propensity in clinical isolates.

This distribution aligns with previous findings reporting a strong predominance of transitions, particularly G → A and C → T changes, in *Mycobacterium tuberculosis*. Such transitions are typically attributed to spontaneous deamination and oxidative DNA damage, both of which are recognized as major mutational forces in GC-rich bacterial genomes. The observed bias toward GC → AT conversions is consistent with the known mutational trend in *M. tuberculosis*, reflecting both intrinsic genome composition and repair dynamics.

Additionally, the high transition rate can be caused by oxidative stress. Antibiotic exposure has been shown to stimulate the production of reactive oxygen species (ROS), which preferentially target guanine—the most oxidation-prone base among the four nucleotides. This leads to the accumulation of G-related mutations, such as G → A or G → C substitutions, as observed in this study. The connection between ROS-mediated DNA damage and antibiotic resistance evolution has been established in various bacteria, including *E. coli*, where increased G:C → A:T transitions occur under antibiotic stress. Thus, the prevalence of guanine-based substitutions in our dataset supports the notion that oxidative damage contributes significantly to mutational dynamics and the adaptive evolution of resistance in *M. tuberculosis* [41]. Moreover, reactive oxygen species (ROS) generate 8-oxoG lesions, which are known to cause G → T mutations, as demonstrated in the mouse germ cell lineage. Consistent with this, regions with elevated recombination rates and single-nucleotide polymorphisms tend to coincide with areas enriched in 8-oxoG, suggesting that this oxidative lesion not only drives G → T substitutions but may also contribute to the increased frequency of recombination events in human genome [42,43].

The well-characterized 81 bp rifampicin resistance-determining region (RRDR) of the *rpoB* gene, mutations at codons 450 (S450L) and 445 (H445Y) are most commonly associated with RIF resistance. Although some reports noted C → T, A → G, and C → A mutations in strains with defective DNA repair systems (e.g., *mutY* variants), the dominant role of oxidative stress and spontaneous base modification appears to be a more universal mechanism influencing the mutation spectrum in both clinical and laboratory strains [20,44].

Overall, the most prevalent nucleotide change in *M. tuberculosis* is the G/C > A/T transition, likely resulting from oxidative damage that the bacterium experiences inside host macrophages. This pattern, first identified in nonhuman primate studies, represents a major mutation type that spreads under relaxed selection in various bacterial species, including *M. tuberculosis*. The genome’s exceptionally high GC content (around 65%) and its abundance of GC-rich homopolymeric regions are thought to further shape and reinforce this mutational bias [45,46].

A notable feature of the mutational spectrum was the strong predominance of G/C. A/T transitions, accounting for nearly half of all observed substitutions. This bias is consistent with previous findings indicating that oxidative stress within host macrophages acts as a dominant mutagenic force in *M. tuberculosis*, preferentially targeting guanine residues [47]. Notably, this bias is stronger in multidrug-resistant isolates compared to drug-susceptible ones, as evidenced by higher overall mutation rates in MDR-associated lineages like Beijing and an enriched spectrum of oxidative transitions during both latent and active phases [48,49]. Mutation bias modeling further shows that such shifts proportionally shape adaptive evolution in MDR strains under antibiotic pressure, amplifying resistance trajectories [50]. Reactive oxygen species (ROS) produced during immune responses can oxidize guanine to 8-oxoguanine, which mispairs with adenine during replication, resulting in characteristic G → T or G → A transitions [51]. The observed pattern of guanine-centered mutations in the current dataset thus reflects an adaptive balance between oxidative DNA damage and the limited capacity of the mycobacterial repair machinery to correct such lesions. Given the exceptionally high GC content of the *M. tuberculosis* genome (approximately 65%), the oxidative environment within macrophages likely intensifies this mutational bias, further influencing the trajectory of resistance evolution.

Interestingly, the conservation of *nucS*, a noncanonical mismatch repair factor, is consistent with its recently described role in limiting transition mutations and maintaining genomic fidelity [52]. Experimental inactivation of the noncanonical mismatch repair gene *nucS* in *Mycobacterium abscessus* has been demonstrated to induce a hypermutator phenotype, characterized by a predominance of transition mutations—patterns that closely mirror those observed in our dataset [53]. However, *nucS* itself did not show any functionally significant mutations in the 175 isolates analyzed in this study. This complete absence of non-synonymous changes indicates that *nucS* is under strong purifying selection and experiences tight functional constraint across the studied population. The few nucleotide substitutions identified were silent and present only in a subset of isolates. Although the S144R substitution is observed relative to the H37Rv reference genome, arginine at this position represents the consensus state among *Mycobacterium* species, indicating that this variant is unlikely to impair NucS function. Therefore, the accumulation of transition mutations in other 3R genes is more likely attributable to disruptions in alternative DNA repair pathways, including downstream effectors or stress-responsive modulators that indirectly compromise mismatch repair efficiency. This interpretation highlights the intricate balance between genomic stability and adaptive mutational plasticity in mycobacteria, enabling enhanced evolvability under antibiotic selective pressures without direct alterations to core mismatch repair components.

Altogether, the findings presented in this study illustrate how the interplay between oxidative stress, impaired repair systems, and selective drug pressure shapes the mutational dynamics of *M. tuberculosis*. The convergence of these processes fosters both short-term adaptation through direct resistance mutations and long-term genomic plasticity, enhancing the bacterium’s ability to survive under therapeutic and immune stress. Given Kazakhstan’s high MDR-TB burden, the identification of such mutational signatures in local isolates emphasizes the urgent need to integrate genomic surveillance into routine diagnostic and public health frameworks. Moreover, early detection of SNPs in 3R genes could serve as predictive markers of hypermutability, offering a new avenue for stratifying patients at risk of resistance amplification during treatment. Future studies combining functional assays with comparative genomics will be crucial to elucidate the mechanistic consequences of these variants and to determine whether targeting DNA repair pathways could represent a viable adjunct strategy for combating drug-resistant tuberculosis.

## 4. Materials and Methods

### 4.1. Data Source

Genomic sequences of *Mycobacterium tuberculosis* were obtained from the Laboratory of Proteomics and Mass Spectrometry at the National Center for Biotechnology of the Republic of Kazakhstan. All sequences were provided in FASTA format and used as the primary data source for downstream bioinformatic analysis.

### 4.2. Genomic Data Processing

Filtering and Preprocessing Raw genomic data were processed using custom Python scripts developed in Python v3.13. The workflow was implemented in Visual Studio Code v1.106.2 and included an initial filtering step to identify sequences containing single-nucleotide polymorphisms (SNPs). Only high-confidence SNPs with sufficient sequence coverage and quality scores were retained for further analysis.

### 4.3. Local Gene Database Construction

A local gene database was constructed to include genes potentially associated with the development of antibiotic resistance in *Mycobacterium tuberculosis*. This database served as a reference for subsequent comparisons and filtering. Genes were selected based on the prior literature, known resistance-associated loci, and recurrent mutation patterns identified in the current dataset.

### 4.4. Bioinformatic Analysis

Filtered sequences were aligned against the local gene database to detect matches indicative of potential antibiotic resistance-related mutations. The resulting dataset was used to quantify the total number of SNPs, transversions, and transitions. Mutation frequencies and types were summarized using Python-based statistical libraries (e.g., pandas, numpy, matplotlib).

### 4.5. Visualization and Statistical Interpretation

To visualize the overall mutation landscape, pie charts were generated to show the proportion of transition and transversion events. The total and unique mutation counts were calculated for each isolate.

A heatmap was then created to display the distribution of mutations across all analyzed *M. tuberculosis* genomes, highlighting patterns of variation among isolates. A three-dimensional (3D) representation of mutation frequency was produced, applying a threshold filter to visualize genes exceeding a defined mutation count.

## Data Availability

The original contributions presented in this study are included in the article. Further inquiries can be directed to the corresponding author.

## Supplementary Materials

The following supporting information can be downloaded at: https://www.mdpi.com/article/10.3390/antibiotics15010026/s1, Supplementary Tables S1–S4.
